# Drs. Catherine and Reginald Hamlin: Pioneers in Obstetric Fistula Repair

**DOI:** 10.7759/cureus.72203

**Published:** 2024-10-23

**Authors:** Nurupa Ramkissoon, Valeria McAllister, Samrawit W Zinabu, Da'Jhai Monroe, Shawn Meepagala, Trinity Gibbs, Hana Gebregziabher, Shaquan Taylor, Miriam B Michael

**Affiliations:** 1 Obstetrics and Gynecology, Howard University Hospital, Washington, DC, USA; 2 Internal Medicine, Howard University College of Medicine, Washington, DC, USA; 3 Internal Medicine, Howard University Hospital, Washington, DC, USA; 4 Anesthesiology, Howard University Hospital, Washington, DC, USA; 5 Pathology, Howard University Hospital, Washington, DC, USA; 6 Obstetrics and Gynecology, Saint Paul's Millennium Medical College, Addis Ababa, ETH; 7 Urology, Howard University Hospital, Washington, DC, USA; 8 Internal Medicine, University of Maryland, Baltimore, USA

**Keywords:** fistula, fistula repair, hamlin, obstructed labor, rectourethral fistula

## Abstract

An obstetric fistula is a serious and debilitating complication of childbirth. It is an abnormal communication between the urinary tract or the gastrointestinal tract and the genital tract, produced by obstetric causes, usually prolonged and obstructed labor. The fetal head presses against the bony pelvis, creating a connection between the genital tract and the bladder or rectum, resulting in incontinence and, frequently, stillbirth. Factors such as early marital age, poverty, and limited access to obstetric care have perpetuated the high incidence of obstetric fistula in Ethiopia. Two Australian physicians, Reginald Hamlin and Catherine Hamlin, arrived in Ethiopia in 1959 and were compelled to address the significantly unmet need for obstetric fistula repair. The Hamlins developed an innovative surgical technique to address this issue. Their commitment extended to the establishment of the Addis Ababa Fistula Repair Hospital, which, over the following decades, provided life-changing reconstructive surgeries to more than 60,000 Ethiopian women.

## Introduction and background

In 1959, a chance encounter with an advertisement in *The Lancet *medical journal led Australian doctors Reginald and Catherine Hamlin to Addis Ababa, Ethiopia [1]. Once there, they encountered a heartbreaking reality: a multitude of women suffering from obstetric fistula, a childbirth injury resulting in urinary or fecal incontinence. Obstetric fistula occurs when the fetus is unable to fit through the birth canal, leading to prolonged, obstructed labor for 4-5 days. Pressure from the baby’s head on the mother’s pelvis over an extended period of time causes tissue necrosis and the formation of an abnormal opening between the vagina and the bladder or rectum. This results in loss of bowel and/or bladder control in the mother and, in 93% of cases, the delivery of a stillborn baby in 93% of cases [2]. Affected women are commonly abandoned by their husbands and deemed responsible for their condition [3]. Ostracized by their families and communities, these women, continuously wet and reeking of urine, sometimes even fecal matter, had virtually no access to treatment [4].

## Review

Early life and career

Dr. Catherine Hamlin, born on January 24, 1924, in Sydney, Australia, graduated from Frensham School, Mittagong, a boarding school in New South Wales. She then went on to attend medical school at the University of Sydney and obtained her medical degree in 1946. She transitioned through two internships at Sydney hospitals and later began her residency in obstetrics and gynecology at Crown Street Women’s Hospital, the largest maternity hospital in New South Wales at the time [5].

**Figure 1:**
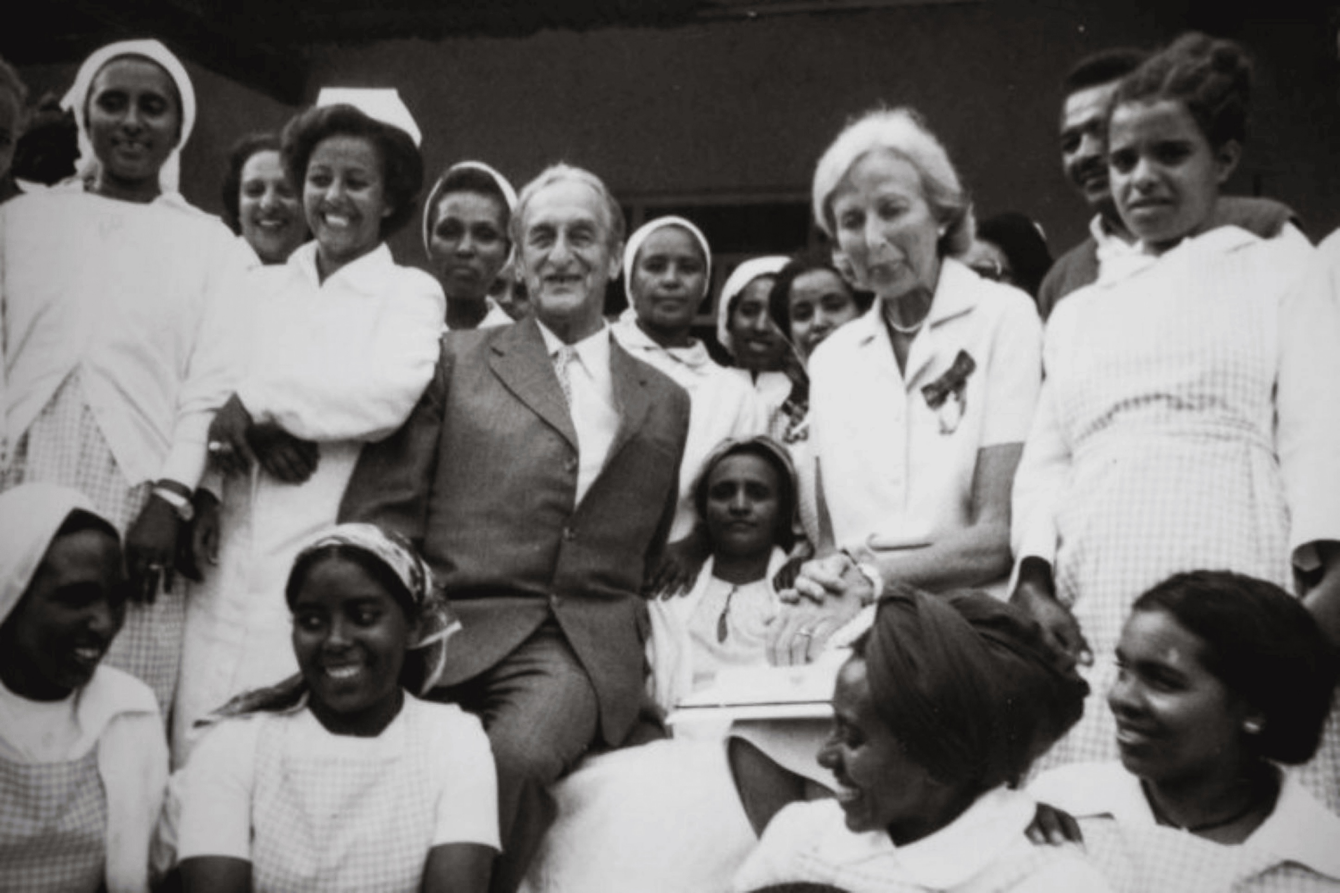
Catherine and Reginald Hamlin with their nursing team. Courtesy of Hamlin Fistula Ethiopia (https://hamlinfistula.org/en/about-us/the-history-of-hamlin/) [6].

Dr. Reginald Hamlin was the medical superintendent at Crown Street who interviewed Catherine for her residency program. He was born on April 21, 1908, in Napier, New Zealand. Reginald began his career as a teacher before obtaining a Master of Arts in History and later attending Otago Medical School in 1941. Prior to his time at Crown Street Women’s Hospital, he worked as a surgeon at Christchurch Hospital and on a New Zealand Navy ship. Catherine and Reginald (Figure 1) married in 1950 and had their only son, Richard, in 1952. In 1959, the couple responded to an advertisement to teach midwifery for three years in Ethiopia. However, once they arrived in Ethiopia, they never left again.

Obstetric fistula and patients in Ethiopia

The large incidence of obstetric fistula in Ethiopia was predominantly influenced by determinants such as residence in rural areas, young age at first childbirth and marriage, delivery location, and educational level [3]. While cesarean delivery can prevent fistula formation, women in rural areas often face significant barriers in accessing qualified physicians or midwives who can perform this procedure. According to Dr. Hamlin, “in the cities in Ethiopia, you never see an obstetric fistula because there are doctors there [2].” In Ethiopia, the fistula patients are usually young, impoverished girls in their early teens with little to no education who were married to farmers [3].

The Hamlins, who had no prior experience with obstetric fistulas due to the rise of safe cesarean sections in developed nations, making fistulas a rarity, embarked on a remarkable journey. Limited by a lack of resources - no ultrasound, generator, or blood bank - they consulted physicians experienced in repairing obstetric fistulas and meticulously studied medical literature, even resorting to 1850s texts, to develop their own surgical technique for fistula repair [5]. This pioneering technique remarkably remains in use today.

"We started with small fistulas," Dr. Hamlin recalled in a 2009 interview, "which any gynecologist can fix without much training, and gradually tackled more difficult ones" [4]. The Hamlins successfully repaired 300 obstetric fistulas in just their first three years. The suffering of these women, however, extended far beyond the physical. Shame, ostracization, and societal stigma became heavy burdens they carried [4]. Even at the hospital, guards initially turned them away due to their lack of funds. Determined to help, Reginald Hamlin would personally seek them out on the hospital grounds, ensuring they received the care they desperately needed.

**Figure 2:**
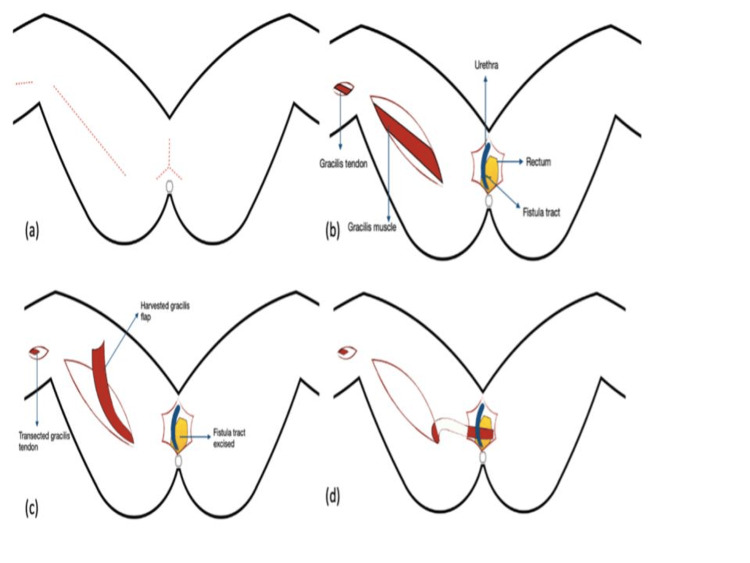
Repair of rectourethral fistula repair using gracilis muscle flap interposition procedure. This figure is taken from Singh et al. [7] with permission from Dr. Prashant Singh (https://doi.org/10.1007/s12262-021-03078-5).

The core procedure consists of the repair of the bladder fistula, followed by the construction of a new urethra. The reinforcement of the newly created urethra is achieved by utilizing the gracilis muscle detached from its natural insertion site and bringing it through a tunnel in the fascia of the upper thigh and the labium to be attached to the anterior lip of the cervix (Figure 2). This method represents an integration of previously developed techniques by Ingelman-Sundberg and Martius, who originally proposed these approaches for other pelvic floor and urological conditions [7].

Legacy

In 1974, amidst a communist revolution, the Hamlins founded the Addis Ababa Fistula Hospital, a haven for these forgotten women [1]. They extended their impact through the establishment of Hamlin Fistula Ethiopia, a healthcare network dedicated to the eradication of obstetric fistulas via the holistic Hamlin Model of Care. This model merges surgical repair, counseling, rehabilitation, and reintegration. The Hamlin Model of Care not only allowed women to regain control of their own bodies but also transformed their lives. Mamitu and Lete were teenagers when they had their fistulas repaired by Dr. Catherine Hamlin. Inspired by the care they received, they both went on to work at Hamlin Fistula Ethiopia. Mamitu was surgically trained by the Hamlins, ultimately becoming one of the leading fistula surgeons in the world. She was even recognized as one of the BBC's top 100 most inspirational women in 2018. Lete, on the other hand, became a staple to the network and ran the outpatient department at Addis Ababa Fistula Hospital. She recounts that the Hamlins treated her as part of their family and watched over her. Both Mamitu and Lete‘s relationship with Dr. Catherine Hamlin developed into a close friendship. Their story is a testament to the Hamlins’ commitment to genuinely care for and better the lives of those they treated as people first, rather than as patients [8].

The Hamlin Model of Care earned Catherine not just lifelong friendships but international recognition as well. She was awarded the 2009 Right Livelihood Award, also known as the “Alternative Nobel Prize,” which honors individuals who provide practical solutions to the most urgent issues confronting humanity. Hamlin was also nominated for the Nobel Peace Prize twice, in 1999 and 2014. In 1995, she was awarded Australia's highest honor: Companion of the Order of Australia. Catherine’s impact on Ethiopia was displayed when she was granted Honorary Ethiopian Citizenship in 2012 by the Ethiopian Government and presented the Eminent Citizen Award in 2019 by Prime Minister Abiy Ahmed [5].

Currently, Hamlin Fistula Ethiopia includes the Addis Ababa Fistula Hospital, five regional fistula hospitals, the Hamlin College of Midwives, 90 midwifery clinics, and the Hamlin Rehabilitation and Reintegration Center [6]. The Hamlin College of Midwives was founded in 2007 to improve the quality and accessibility of maternal care. On the other hand, the Hamlin Rehabilitation and Reintegration Center fistula supports patients with severe injuries in their healing process while providing counseling, literacy education, and vocational and life skills training, assisting them in reintegrating into their communities and securing employment. Drs. Catherine and Reginald Hamlin also published and taught extensively, developing a systematic approach to treating this complex injury [9,10]. Over the decades, Hamlin Fistula Ethiopia has successfully provided reconstructive surgery to over 70,000 Ethiopian women.

## Conclusions

While obstetric fistula is still a large issue in resource-limited locations like Ethiopia, Drs. Catherine and Reginald Hamlin have greatly reformed the treatment of women suffering from this condition. The Hamlins’ legacy has evolved from focusing solely on repair and treatment to prioritizing prevention with a goal of placing a well-trained midwife in every village, aligning with their vision to eradicate obstetric fistulas. Both their surgical technique and holistic model of care have altered the lives of countless women and enriched the field of obstetrics and gynecology. These transformative procedures and rehabilitation programs have renewed the lives of tens of thousands of Ethiopian women, reinstating their sense of confidence and dignity while restoring their place in society. The Hamlins serve as an example of selflessness and dedication to the betterment of women’s health. Dr. Catherine Hamlin continued her work well into her 90s until her passing, a testament to her unwavering commitment. Through our review, we aimed to display the profound impact that the Hamlins made on the management of obstetric fistulas in Ethiopia.
